# A Preliminary Mechanics-Informed Machine Learning Framework for Objective Assessment of Parkinson’s Disease and Rehabilitation Outcomes

**DOI:** 10.3390/diagnostics15222855

**Published:** 2025-11-12

**Authors:** Amirali Hanifi, Roozbeh Abedini-Nassab, Mohammed N. Ashtiani

**Affiliations:** 1 Faculty of Mechanical Engineering, Tarbiat Modares University, Tehran 14115-111, Iran; amiralihanifi80@gmail.com; 2 Faculty of Medical Sciences, Tarbiat Modares University, Tehran 14115-111, Iran; mnashtiani@modares.ac.ir

**Keywords:** Parkinson’s disease, ground reaction forces, postural mechanics, biomechanical signal processing, center of pressure, unsupervised learning, rehabilitation outcome assessment

## Abstract

**Background/Objectives:** Non-invasive methods for evaluating rehabilitation outcomes in Parkinson’s disease (PD) remain limited. This preliminary study proposes a mechanics-informed machine learning (ML) framework integrating force-plate data with dimensionality reduction, clustering, and statistical analysis to objectively assess motor control and the effects of a targeted intervention. **Methods:** Twelve PD patients were randomly assigned to a PD control group performing standard exercises or an intervention group incorporating additional transverse-plane trunk motion exercises for 10 weeks. Ground reaction forces and center of pressure (COP) signals were recorded pre- and post-intervention using a force plate, alongside data from six healthy individuals as a benchmark. Features related to postural sway and COP dynamics were extracted and refined using Forward Feature Selection. Dimensionality reduction (t-SNE) and unsupervised clustering (K-means) identified group-level patterns. SHAP values and Cohen’s d quantified feature importance and effect size. Clustering robustness was assessed with bootstrapping, nested cross-validation, and permutation testing. **Results:** K-means clustering revealed clear pre/post-intervention separation in five of six intervention patients, with post-intervention states shifting toward the control cluster. Clustering showed strong performance (Silhouette 0.77–0.79; Calinski–Harabasz 100.8–184.9; Davies–Bouldin 0.29–0.45). The most predictive features (RMS-SML and PL-SAP) showed large effect sizes (Cohen’s d = –12.1 and –4.53, respectively) distinguishing PD patients from healthy controls. Traditional statistical tests (e.g., ANOVA) failed to detect within-group changes (*p* > 0.05), but ML-based methods captured subtle, nonlinear postural adaptations. **Conclusions:** This preliminary mechanics-informed ML framework detects PD-related motor deficits and rehabilitation-induced improvements using force-plate data, warranting validation in larger cohorts.

## 1. Introduction

Parkinson’s disease (PD) is a chronic, progressive neurodegenerative disorder [1] that predominantly impairs motor functions, including movement, balance, and coordination [2]. The degeneration of dopamine-producing neurons leads to disruptions in neural circuits responsible for motor control [3]. Currently, approximately six million people worldwide are living with PD, a number expected to double to 12 million by 2040 [4]. Diagnosing PD and monitoring its progression remain significant challenges, as clinical evaluations of motor symptoms are subjective and difficult to quantify consistently [5]. These challenges highlight the need for objective biomechanical measurements to assess motor deficits more accurately.

While pharmacological treatments such as Levodopa are commonly used in PD management, they may cause side effects including nausea, dyskinesia, and hallucinations, particularly in later stages [6]. Surgical options such as deep brain stimulation (DBS) offer benefits but are invasive and costly [7]. Physiotherapy interventions (PIs), on the other hand, provide a safer, personalized alternative that can improve balance, strength, and flexibility in individuals with PD [8]. However, outcomes across studies remain inconsistent. For example, Freidle et al. [9] reported no significant improvement in balance or motor function following a 10-week targeted balance training intervention. Rabini et al. [10] reported improvements in balance but only stabilization (not improvement) in upper and lower limb mobility. The clinical effects are inconsistently reported, and outcomes often rely on subjective scales that may fail to capture subtle biomechanical improvements [11,12]. Given these limitations, developing objective and clinically meaningful assessment tools to evaluate intervention outcomes is a key goal in modern PD care [13,14]. To address this need and considering the importance of balance control and postural adjustments in PD, we designed an intervention focused on Transverse Plane Exercise Intervention (TPEI). These exercises target neuromechanical coordination during movements, which are often important in PD. We hypothesized that transverse plane exercises would improve dynamic postural control by enhancing torque regulation and modulating feedback delays in the neuromechanical system.

The analysis of the PI outcomes in PD is challenging due to the high-dimensional nature of biomechanical data and the subtlety of motor changes [15]. Machine learning (ML) approaches have been increasingly adopted to address these complexities. Supervised analysis approaches are the most common, involving feature extraction from speech, handwriting, movement, and brain scans, followed by classification using models such as Support Vector Machine (SVM), K-Nearest Neighbor (KNN), logistic regression (LR) or gradient boosting. For instance, high diagnostic accuracy was achieved with only seven acoustic features in speech analysis using supervised ML, which works better than traditional statistics [16], and voice signal classification using KNN and Feed-Forward Neural Networks (FNN) has also shown strong results [17]. Deep learning applied to magnetic resonance images (MRI) [18] and brain morphology [19] has revealed neuroanatomical changes related to PD severity, while random forest (RF) has been used to analyze large-scale clinical datasets [20]. In gait analysis, features such as step velocity and variability were selected using recursive feature elimination [21], while center-of-pressure (COP) sway patterns from force-plate data were classified using support vector machines (SVM) [22]. Unsupervised and self-supervised methods are less common but emerging. For example, one study categorized speech patterns through hierarchical clustering to improve UPDRS rating consistency [23]. Also, self-supervised speech models such as Wav2Vec along with supervised contrastive learning, have shown promising results for interpretable early detection of PD on unlabeled datasets [24].

Combining a wearable sensor equipped with an accelerometer and a CNN, the severity of PD can be evaluated based on tremor intensity [25]. Given the limitations of traditional statistical methods for large datasets, dimensionality reduction techniques such as Principal Component Analysis (PCA) are commonly applied [26]. In electroencephalogram (EEG) analysis, PCA followed by SVM has enabled early PD detection [27]. For diagnosing PD based on electromyography (EMG) signals, models such as LR, Naïve Bayes, and Artificial Neural Networks (ANN) have also been explored [28]. However, these studies often rely on labeled datasets and report classification metrics, which are not the focus of our exploratory investigation. A comparison of previous works is listed in Table 1.

The current study proposes a mechanics-informed machine learning approach using force-plate-derived kinetic data. By combining unsupervised clustering (e.g., K-means) and dimensionality reduction (t-SNE) with statistical analysis, this approach enables the exploration of motor patterns and the identification of subtle biomechanical changes associated with PD and TPEI outcomes. Specifically, we aim to (1) examine the capability of this framework for PD diagnosis, (2) evaluate TPEI effects on motor and balance performance, and (3) assess ML models for tracking pre/post-intervention changes. Our main goal is to develop and validate the biomechanical analysis method using TPEI as a test case and not to prove the clinical efficacy of TPEI itself.

We focused on three clinically relevant force-plate features. PL-SAP (Path Length—Standing, Anterior–Posterior) reflects the total center of pressure (COP) displacement in the sagittal plane during quiet standing. Increased values of PL-SAP may indicate instability and elevated energy expenditure, consistent with known postural deficits in PD. RMS-SML (Root Mean Square—Standing, Medial-Lateral) quantifies sway variability by measuring the RMS of COP displacement in the frontal plane. LDisp-Gait (COP Displacement during Gait Initiation—Loading Phase) captures medial-lateral COP movement during early gait initiation, which is often reduced in patients with PD. While force plate features offer objective insight into balance and motor control, their clinical interpretation requires nuance, especially in PD. In general, greater COP displacement, path length, and sway area are linked with reduced postural stability and increased fall risk. However, in Parkinson’s disease, extremely small sway or minimal movement may also reflect pathological rigidity or freezing, rather than good stability. Therefore, changes in these features should be interpreted in the context of motor symptoms, not just in terms of more or less movement. To ensure data reliability, we applied strict inclusion and exclusion criteria and standardized testing conditions. This study aims to identify the most informative biomechanical markers of PD using a simple, low-cost assessment approach.

Unlike prior supervised studies [17,19], our framework integrates unsupervised, mechanics-informed analysis of COP data to detect intervention effects without labeled inputs. Because there is no universally accepted clinical threshold for quantifying physiotherapy-induced balance improvement in PD, assigning objective labels was not feasible. Therefore, we chose an unsupervised approach to uncover latent biomechanical patterns in the data without relying on potentially biased or subjective classification. Furthermore, our methodology supports robust validation through nested cross-validation, bootstrapping, and permutation testing, which collectively help reduce the risks of overfitting, information leakage, and false-positive patterns [38,39]. To improve interpretability and clinical relevance, we used the SHAP values and Cohen’s d effect sizes to highlight the most influential features and quantify the magnitude of intervention-related changes. Notably, while conventional statistical analyses often fail to detect significant changes post-intervention, our AI-based clustering suggested clear and meaningful pattern changes, demonstrating superior sensitivity to subtle improvements in balance and motor control. Unlike traditional methods that often rely on specialized and costly equipment and may miss subtle patient changes [40,41], this approach offers a scalable and clinician-friendly alternative. This mechanics-informed integration provides a physics-grounded complement to data-driven approaches, promoting clinical interpretability. Additionally, it adds to the growing body of evidence supporting the use of biomechanical analysis as a reliable tool for monitoring motor and balance impairments in PD, with potential to inform future clinical practice. The manuscript is organized as follows: Section 2 describes participants, intervention methods, data acquisition and analysis, Section 3 presents results, and Section 4 and Section 5 provide discussion and conclusions.

## 2. Methods

### 2.1. Participants

In this study, a total of 18 participants were included: 12 individuals with PD (6 in the PD intervention group receiving TPEI exercises and 6 in the PD control group) and 6 healthy individuals in the healthy control group. The goal is to compare the behavior of PD patients receiving TPEI with that of the other PD patients, as well as with healthy individuals, to observe the effects of TPEI. Patients were referred by a neurologist and a neurological physiotherapist (Iran University of Medical Sciences, Firoozgar Hospital and Movement Disorders Laboratory, Tarbiat Modares University) to participate in the study. Eligibility was determined based on the following inclusion criteria: (i) a confirmed diagnosis of idiopathic Parkinson’s disease based on the UK Brain Bank Criteria, (ii) Hoehn and Yahr (H&Y) stage between 2 and 3, (iii) age between 35 and 80 years, (iv) body mass index (BMI) between 18.5 and 30, (v) ability to walk unaided, (vi) no history of neurological conditions other than PD, (vii) absence of metabolic disorders such as diabetes or osteoporosis, and (viii) no musculoskeletal disorders apart from PD-related symptoms, to avoid biomechanical confounds that could affect the mechanics-informed analysis of force-plate data.

Of the 16 PD patients initially enrolled, four withdrew, leaving 12 PD patients who completed the study. Eligibility was confirmed through a comprehensive physiotherapy evaluation during the first visit. Participants received detailed verbal and written information outlining the study’s objectives, procedures, and exercise protocol.

The average Hoehn and Yahr (H&Y) stage was 2.4 ± 0.41 in the PD control group and 2.083 ± 0.2 in the PD intervention group, indicating that participants were in the mild-to-moderate stage of Parkinson’s disease. The between-group difference was statistically significant (*p* = 0.0408).

**Figure 1:**
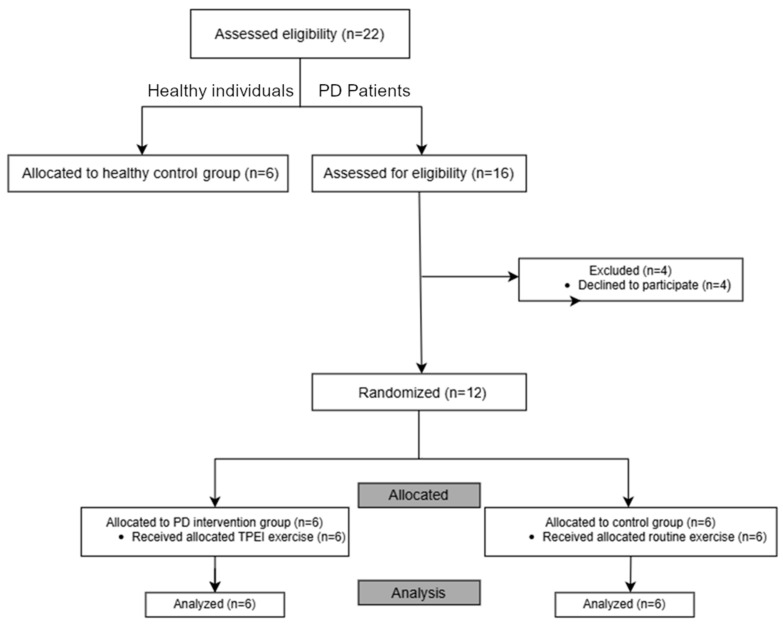
Flowchart illustrating the distribution of participants.

Additionally, six healthy individuals were recruited as a healthy control group to assess diagnostic capabilities and intervention effectiveness. Inclusion criteria for healthy volunteers included: (i) no symptoms of PD, (ii) ability to stand and walk unassisted, and (iii) no musculoskeletal issues. The distribution of participants is shown in Figure 1. Demography of participants is presented in Table 2 with a summarization in Table 3. Although the cohort size is relatively small, reflecting the strict inclusion criteria and the demanding experimental setup, this study was designed as a preliminary proof-of-concept investigation.

Written informed consent was obtained. Ethical approval was granted by the university’s medical research ethics board (approval code IR.MODARES.REC.1400.098, Date: 26 June 2021) and the study was registered as a clinical trial (registration code: IRCT20210516051322N1, Date: 25 July 2021).

### 2.2. Study Design, Routine Activities, and Intervention Protocol

This study follows a randomized single-blind design, where the analyst is unaware of the participants’ group assignments. To maintain this blinding, each participant was given a neutral code that did not reveal the PD intervention group to which he or she was assigned. This approach helps reduce potential bias during the analysis. Group assignment was performed using the sealed envelope method. Once patients met the inclusion criteria, a blinded laboratory technician who was not involved in the study randomly selected an envelope, and the patient was then assigned to either the control or exercise (PD intervention) group. While the researchers were aware of the group allocations, this information was kept hidden from the participants to maintain partial blinding. During data analysis, the ground-truth labels (patient identifiers) were withheld from the system, ensuring that the model operated entirely on unlabeled data. The labels were added only at the final stage to the plotted graphs to facilitate interpretation. Participants were unaware that another group existed.

The PD control group performed routine activities and usual care with exercises such as a 15 min warm-up, walking and stretching three times a week for 10 consecutive weeks. Each session lasted totally 45 min. Approximately 20 percent was added to the number of movements in each set every two weeks to have a progressive design. The PD intervention group performed a set of warm-ups, walking and stretching for 15 min, then transverse plane exercises for 30 min (overall 45 min) to improve gait and posture control by affecting the trunk rotator and axial muscles, three times a week for 10 consecutive weeks. Approximately, 20 percent was added to the number of movements in each set every two weeks. They engaged in trunk rotational exercises targeting key lower trunk muscles (external/internal obliques, rectus abdominis, erector spinae). These muscles, often impaired in Parkinson’s disease (PD), are critical for trunk stability and postural control. Strengthening them may improve upper-body sway management and gait initiation via enhanced pelvic and trunk coordination. Compliance was monitored via weekly video calls and submitted recordings, with an in-person home visit at week 5 to assess exercise performance.

Participants completed a structured 10-week proprioception-focused physical intervention program, with sessions held twice weekly for 45 min. Each session included 10 proprioceptive stations designed to challenge biomechanical balance control and neuromechanical coordination: (1) tandem walking, (2) toe walking, (3) heel walking, (4) single-leg standing (eyes open), (5) single-leg standing (eyes closed), (6) standing on foam (eyes open), (7) standing on foam (eyes closed), (8) standing on astroturf (eyes closed), (9) standing on marbles (7 mm) (eyes closed), and (10) standing on a BOSU ball (eyes open).

### 2.3. Methodologies

#### 2.3.1. Biomechanical Data Acquisition

**Figure 2:**
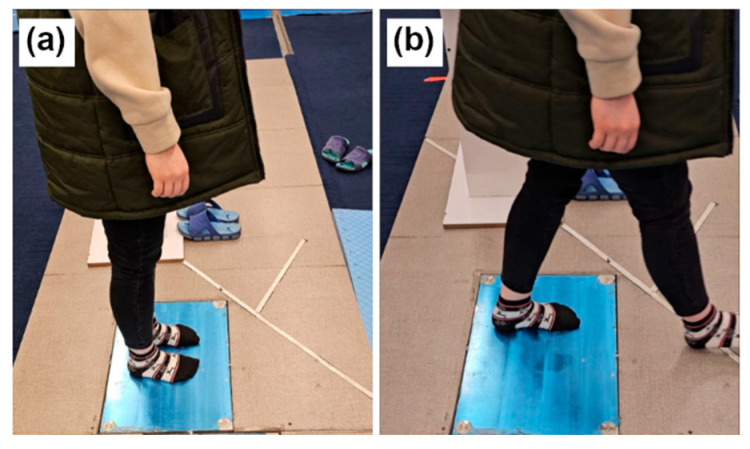
The force-plate experiments. (**a**) Standing. (**b**) Walking.

Two biomechanical tests were conducted in the Physiotherapy Laboratory at Tarbiat Modares University using a Kistler 3D force-plate (Figure 2): (i) standing test, in which participants stood on the plate for 30 s while focusing on a fixed point and (ii) gait initiation test, in which participants stood still for 3 s, then initiated gait upon a verbal cue. The force-plate recorded data at 1000 Hz, capturing ground reaction forces (GRFs), moments, and center of pressure (COP) across all three axes (x, y, z), representing primary kinetic and postural control variables essential in biomechanical analysis.

To bridge biomechanics with machine learning, mechanics-informed features were derived based on physical interpretations of balance and motion. Primary signals recorded by the device were insufficient for comprehensive analysis. Therefore, features and variables grounded in biomechanical principles describing the dynamics and balance of the patients were extracted.

#### 2.3.2. Signal Pre-Processing and Data Integration

The continuous-time signals of ground reaction forces, moments, and center of pressure (COP) were first trimmed to remove transient states at the beginning and end of each trial. The signals were then filtered using a low-pass filter to eliminate high-frequency noise. From the processed signals, biomechanical variables describing the magnitude, displacement, and velocity of oscillations were calculated over the duration of the experiment. All extracted features were subsequently standardized before feature selection, dimensionality reduction, and clustering, ensuring comparable scaling across participants and measurement units.

Initially, features were extracted from continuous-time signals, forming five data frames: standard deviation of force, moments, and center of pressure (FMCOP); features of standing (mediolateral); features of standing (anterior–posterior); features of ground reaction force ($F_{z}$); and features of gait. When analyzed separately, these showed limited structure. Therefore, the dataframes were merged into a comprehensive feature matrix, including demographic variables (age, gender, BMI), which produced more informative patterns for analysis.

#### 2.3.3. Feature Extraction

From the preprocessed and filtered signals, mechanics-informed features were extracted to quantify participants’ balance control and gait initiation mechanics. These features were derived based on biomechanical principles describing the dynamics of postural stability and neuromechanical control.

From the standing test, Sway Area, Path Length, Variance, Root Mean Square, and Displacement Velocity of COP were extracted. Variables were calculated for both the mediolateral and anterior–posterior axes.

From the gait initiation test, displacement and velocity of COP in the mediolateral and anterior–posterior directions during the loading and unloading phases were extracted. The tests were conducted before and after the intervention.

#### 2.3.4. Data Analysis

Several feature selection algorithms were evaluated to identify the most informative biomechanical features. Forward Feature Selection (FFS) was ultimately chosen due to its computational efficiency and interpretability, particularly suitable for small, high-dimensional datasets (e.g., 18 participants, >30 features), starting with an empty subset and adding features one by one based on model performance evaluation. FFS utilized LR and KNN classifiers to evaluate candidate feature subsets based on classification accuracy. To prevent the risk of overfitting, model performance during FFS was assessed using nested cross-validation. To further validate the results, bootstrap resampling (1000 iterations) and permutation testing were applied. These validation strategies were used to compute performance metrics (e.g., accuracy, F1 score, AUC) with corresponding confidence intervals and *p*-values to determine statistical significance and generalizability. This rigorous approach ensured robust feature selection despite the relatively small sample size. FFS was chosen for its efficiency and interpretability in small, high-dimensional datasets. Alternative methods such as Recursive Feature Elimination (RFE) and Exhaustive Feature Selection (EFS) were evaluated but found computationally less practical for this dataset. Similarly, EFS was found impractical because of its combinatorial complexity. Finally, three features were suggested by FFS to form a new feature vector.

After supervised feature selection, the reduced feature set was analyzed using unsupervised clustering and dimensionality reduction techniques to explore the intrinsic structure of the data without relying on class labels. Specifically, t-SNE was applied for dimensionality reduction. PCA was also tested but resulted in overlapping projections that failed to reveal meaningful separations. Given the nonlinear relationships among biomechanical features, t-SNE was selected as it better preserves local neighborhood structures and captures nonlinear patterns in high-dimensional data. K-means clustering was then used for unsupervised grouping, in a purely exploratory, label-free manner. Other clustering algorithms (e.g., Density-Based Spatial Clustering of Applications with Noise (DBSCAN) and hierarchical clustering) were also tested but did not yield clearer or more interpretable results. For instance, DBSCAN, which identifies clusters based on local point density and can detect non-spherical structures as well as outliers, was implemented to assess the robustness of the clustering results. DBSCAN parameters (eps = 0.687, min_samples = 2) were optimized, but the resulting clusters did not offer clearer or more interpretable patterns compared to those from K-means. Based on these comparative assessments, K-means was retained as the most interpretable and parsimonious method for the given dataset and exploratory purpose.

Pairwise similarities in high and low-dimensional space were calculated using Equations (1) and (2), respectively.

(1) $$P_{ij}=\frac{\exp\left(-\frac{{∥x_{i}\;-\;x_{j}∥}^{2}}{2{\sigma_{i}}^{2}}\right)}{\sum_{k\neq l}\exp\left(-\frac{{∥x_{k}\;-\;x_{l}∥}^{2}}{2{\sigma_{k}}^{2}}\right)}$$

(2) $$Q_{ij}=\frac{{\left(1+{∥y_{i}-y_{j}∥}^{2}\right)}^{-1}}{\sum_{k\neq l}{\left(1+{∥y_{k}-y_{l}∥}^{2}\right)}^{-1}}$$

where $x_{j}$ and $x_{j}$ represent data points in the original high-dimensional feature space, while $y_{i}$ and $y_{j}$ denote their corresponding representations in the reduced low-dimensional space. The term $\sigma_{i}$ is the bandwidth of the Gaussian kernel centered at $x_{i}$, which controls the local neighborhood size around each data point. $P_{ij}$ in Equation (1) defines the conditional probability that point $x_{i}$ would choose $x_{j}$ as its neighbor, assuming a Gaussian distribution of distances in the high-dimensional space. $Q_{ij}$ in Equation (2) defines a similar probability distribution in the low-dimensional space, computed using a Student’s t distribution with one degree of freedom to allow heavier tails and prevent crowding. The cost function for optimization is the Kullback–Leibler (KL) divergence, which should be minimized. This function measures the relative entropy between distributions P and Q (Equation (3)). Minimizing this divergence ensures that the pairwise similarities in the low-dimensional embedding best preserve the neighborhood structure of the original data. In this study, t-SNE was used to project the selected biomechanical features into a two-dimensional space for visualization and exploratory clustering.

(3) $$KL\left(P∥Q\right)=\sum_{i\neq j}P_{ij}\;log\frac{P_{ij}}{Q_{ij}}$$

Healthy individuals were included in this study to evaluate differences between them and patients, and to assess whether the intervention led to improvements. Data analyses focused on unsupervised and exploratory methods rather than classification. Nested cross-validation, bootstrapping, and permutation tests were applied to ensure the robustness and reliability of the findings. We reported the metrics of each model with mean ± standard deviation, confidence intervals, and *p*-values to assess whether the performance is random or not.

In addition to nested cross-validation, bootstrap resampling, and permutation testing to ensure robustness and to prevent overfitting, we re-analyzed our data to validate the clustering metrics on unseen data as well. Also, to quantify whether the changes in variables and metrics correspond to clinically meaningful differences, we calculated Cohen’s d effect sizes for center of pressure characteristics under pre- and post-intervention conditions for both groups. Ultimately, to highlight which features most influence classification for better interpretability, we applied SHAP as an explainability method on the selected features and our classification model to ensure that the findings reflect the actual decision-making process of the model.

## 3. Results

Complete demographic characteristics for the three groups are presented in Table 2. No statistically significant differences were found between the PD control and PD TPEI groups in Hoehn and Yahr (H&Y) stage (*p* = 0.219) or BMI (*p* = 0.605), indicating that disease severity and body composition were comparable at baseline. The slight difference in age between the PD groups (*p* = 0.056) is due to the younger age of the TPEI group. As expected, both PD groups differed markedly from the healthy control group in age (*p* < 0.001), reflecting the age-related prevalence of Parkinson’s disease. Sex distribution was approximately balanced across all groups. These results confirm that the two PD subgroups were demographically homogeneous and comparable in key clinical parameters prior to intervention. Therefore, the post-intervention differences observed in the biomechanical and clustering analyses can be attributed primarily to the physiotherapy-based exercise intervention rather than demographic confounders such as age, sex, or disease stage.

To identify the most mechanically informative features, FFS was applied using LR and KNN classifiers. Table 4 lists the most predictive features selected by the models. The selected features represent quantifiable outcomes of the person balance and gait mechanics, derived from Newtonian principles applied to COP motion and ground reaction forces.

To assess the discriminative power of the selected features (RMS-SML, PL-SAP, LDisp-GAIT), a logistic regression model was trained using these three variables to predict group membership (PD vs. healthy). The model achieved a McFadden pseudo-R^2^ of 0.30, indicating that the selected feature vector explains approximately 30% of the variance in group classification and captures meaningful structure for subsequent clustering.

These features are mechanics-informed indicators of human balance control, reflecting how individuals manage ground reaction forces and COP excursions to maintain their stability.

### 3.1. Mechanical Impact of the Intervention

**Figure 3:**
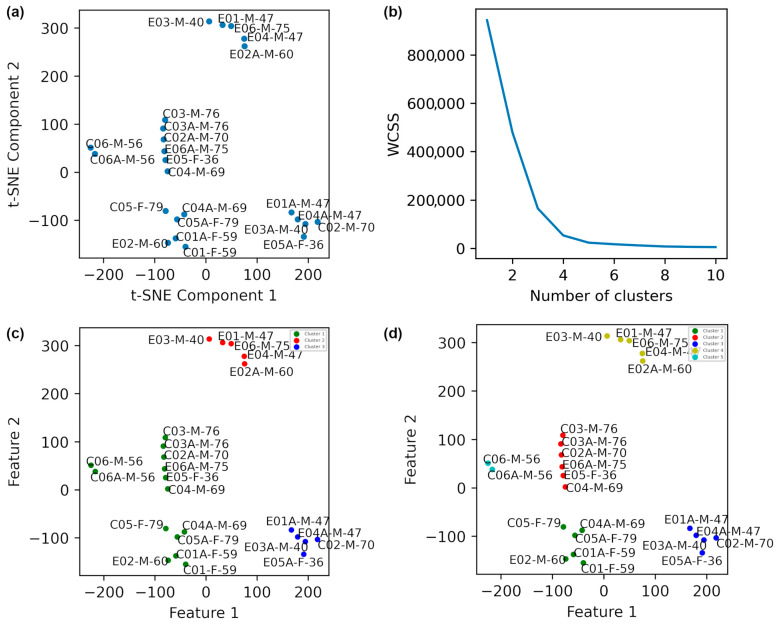
Dimensionality reduction in data obtained from the patients using t-SNE with a perplexity of 2 is illustrated. (**a**) The plot before clustering. (**b**) The elbow method plot. (**c**) K-Means clustering with k = 3. (**d**) K-Means clustering with k = 5. In the labels, E, C, A, F, and M denote with intervention exercise, control, after exercise, female, and male, respectively. The final digits indicate the age of the participant.

To facilitate more comprehensive analysis and visualization, the selected feature vector (SFV) was reduced to two dimensions using t-SNE with a Perplexity of 2. The resulting 2D projection is shown in Figure 3a. It should be noted that t-SNE is exploratory and not directly reproducible. In this study, it was used for visualization and a better understanding of the data structure (although for t-SNE, we used “random_state = 42” and declared specific “perplexity” values to prevent random initialization and generation of stochastic results).

The Elbow method was employed to estimate the optimal number of clusters, using the within-cluster sum of squares (WCSS) as the evaluation criterion (Equation (4)). The Elbow plot in Figure 3b shows clear changes in slope at k = 3 and k = 5, indicating those as plausible numbers of clusters.

(4) $$WCSS=\sum_{j=1}^{k}\sum_{x_{i}\in C_{j}}{∥x_{i}-\mu_{j}∥}^{2}$$

This measure detects how biomechanically similar patients are in their balance control characteristics. K-means clustering was subsequently applied for both k = 3 and k = 5. With k = 3 (Figure 3c), patients 1, 3, 4, and 5 from the PD intervention group showed clear pre/post intervention separation, whereas the PD control group largely remained in a single cluster.

When k = 5 was used (Figure 3d), all TPEI patients showed distinct cluster shifts (i.e., significant mechanical adaptations) between pre- and post-intervention states. Specifically, patients 1, 3, and 4 transitioned from one cluster (red) to another (blue), and patients 2, 5, and 6 exhibited similar patterns. The PD control group remained clustered with minimal change, indicating limited or no impact from the intervention. These findings suggest that the intervention positively affected the neuromechanical control system of the patients, helping them to better modulate forces and COP trajectories.

To further characterize the identified clusters, we calculated the descriptive statistics, including mean, variance, and standard deviation, for each cluster. Table 5 summarizes the results for each cluster for the cases of k = 3 and k = 5, separately. These statistical metrics provide additional insights into the internal homogeneity and distinctiveness of the groups formed by the clustering process.

### 3.2. Mechanical Convergence Toward Healthy Dynamics

**Figure 4:**
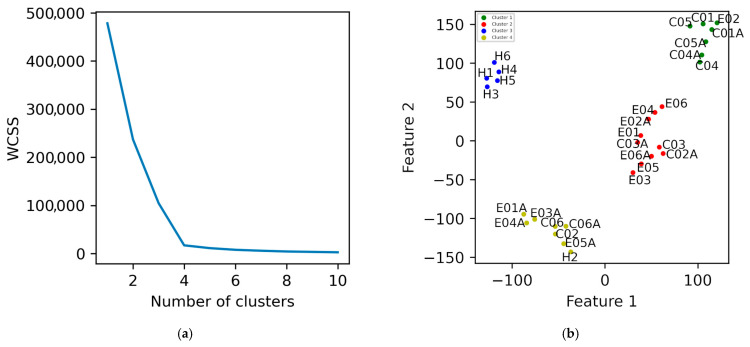
Dimensionality reduction in data obtained from the healthy individuals and patients using t-SNE with a perplexity of 3 is illustrated. (**a**) The elbow plot. (**b**) K-Means clustering with k = 4. The labels E, C, A, and H in the names denote with intervention exercise, control, after exercise, and healthy individuals, respectively.

To determine if the intervention brought patients closer to a healthy postural control pattern, healthy individuals were added to the dataset. SFV was recomputed and reduced using t-SNE (perplexity = 3). Figure 4a shows the Elbow plot, suggesting optimal k values of 2, 3, and 4.

In the two-cluster solution (Supplementary Figure S1a), post-intervention states of TPEI patients 1, 3, 4, and 5 clustered with healthy individuals (green), indicating convergence toward healthy biomechanical balance, while pre-intervention states remained separated in a different cluster (red). The PD control group did not show any similar change. With three clusters (supplementary Figure S1b), a similar pattern persisted—TPEI patients transitioned from the pre-intervention (green) to post-intervention (blue) clusters. Finally, using four clusters (Figure 4b), patient 2 also exhibited clear pre/post separation, indicating that five of six TPEI patients showed a mechanical shift post-exercise measurably toward the healthy cluster. The evidence of changes in force distributions and COP modulation patterns suggests that the exercises improved the ability of the patients to stabilize themselves mechanically.

We also calculated the descriptive statistics of each cluster in Figure 4b and Supplementary Figure S1, and the results are presented in Table 6.

### 3.3. Diagnostic Approach

**Figure 5:**
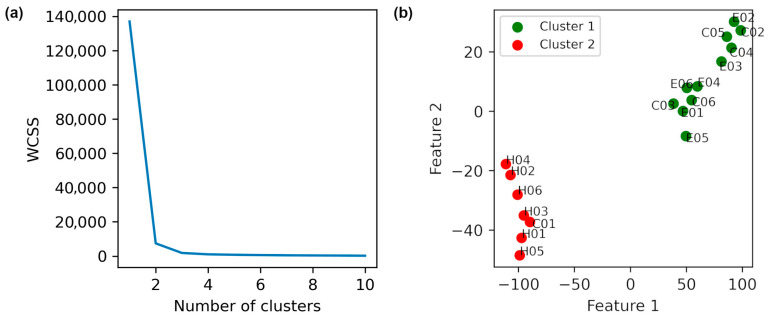
Distinguishing healthy individuals from patients based on t-SNE method with a perplexity of 3 is shown. (**a**) Elbow method plot. (**b**) K-Means clustering with k = 2. The labels E, C, and H in the names denote with intervention exercise, control, and healthy individuals, respectively.

The data of healthy individuals was obtained from the force-plate signals, including forces, moments, and center of pressure values during 30 s. A combined dataset was created from force-plate signals obtained from healthy individuals and patients. Standard deviation (SD) values for each feature were extracted, and t-SNE was applied (perplexity = 3). The Elbow plot (Figure 5a) supported k = 2, and K-Means clustering (Figure 5b) clearly separated patients and healthy controls into distinct clusters. This finding validates the hypothesis that Parkinson’s pathology is revealed in altered biomechanical stability patterns.

The descriptive statistics of the clusters obtained in Figure 5 (Table 7) provide additional evidence that the identified groups are internally consistent and externally distinct, further supporting the separation between patients and healthy controls.

The quality of clustering was evaluated using three indices. The Silhouette index measures how similar each point is to its own cluster compared to other clusters, ranging from −1 to +1; higher values indicate better cohesion within clusters. The Davies–Bouldin index assesses cluster similarity and compactness; lower values indicate more distinct and tighter clusters. Finally, the Calinski–Harabasz index evaluates the ratio of between-cluster dispersion to within-cluster dispersion, with higher values reflecting denser clusters and greater separation. These metrics provide descriptive evidence of meaningful structure in the data, supporting the observation of post-intervention changes. The Silhouette, Davies–Bouldin, and Calinski–Harabasz indices, calculated on unseen data through bootstrapping (Table 8), show narrow 95% confidence intervals, indicating that the observed clustering is robust and not random.

### 3.4. Pre-Intervention State Analysis

**Figure 6:**
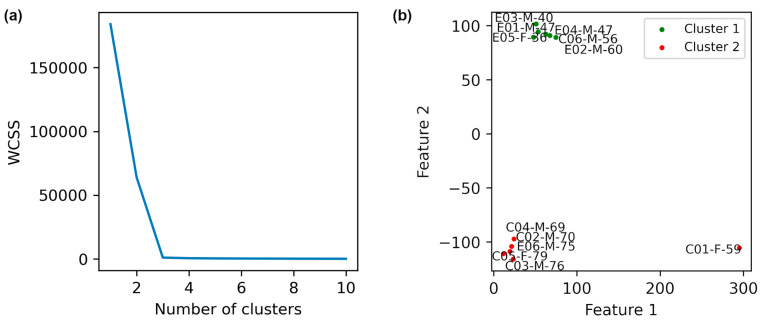
Evaluation of patient conditions before the exercise is depicted. (**a**) Elbow method plot. (**b**) K-Means clustering of pre-intervention data. Clustering was performed based on t-SNE with perplexity = 1. The labels E, C, A, and H in the names denote with intervention exercise, control, after exercise, and healthy individuals, respectively.

To study baseline mechanical heterogeneity among patients (i.e., before any exercise), we clustered the data of their pre-intervention states. The t-SNE result for a perplexity of 1, indicating 2 clusters according to the elbow method in Figure 6a, is plotted in Figure 6b. Based on this figure, the average age of individuals in the green cluster is lower than that of those in the red cluster.

### 3.5. Clinically Meaningful Differences

**Figure 7:**
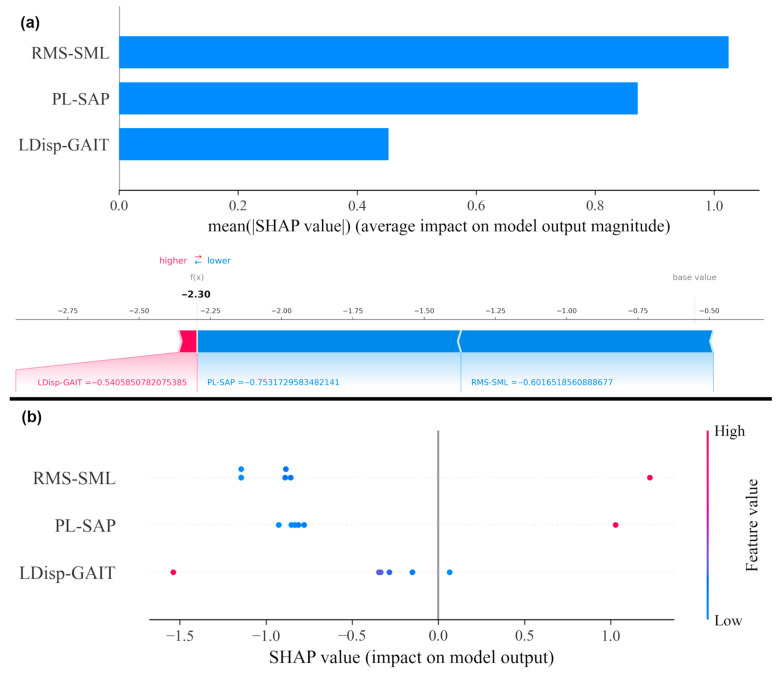
SHAP analysis on COP metrics. (**a**) Mean SHAP values for features, indicating their relative importance in the model. (**b**) SHAP dependence plot for features, showing how individual feature values influence model output.

The results of Cohen’s d effect sizes and SHAP are reported in Table 9 and Figure 7, respectively. Figure 7a shows the mean SHAP values for features, indicating their relative importance in distinguishing PD patients from healthy individuals. Panel (b) shows SHAP dependence plots, illustrating how changes in individual feature values influence model predictions. Both analyses consistently showed that RMS-SML and PL-SAP contribute the most to distinguishing PD patients from healthy individuals. This converges with the effect size analysis and reinforces the clinical significance of these features in describing improvements in postural stability. This alignment and convergence between machine learning explanations and clinical acceptability strengthens confidence in the validity of the model.

Static COP features (RMS-SML and PL-SAP) provided clearer separation between PD patients and healthy individuals, suggesting greater diagnostic utility. In contrast, the dynamic COP feature (LDisp-GAIT) was more sensitive to post-intervention changes, indicating that static and dynamic balance measures may serve complementary roles in assessment and rehabilitation.

To statistically interpret the clinical meaning of these cluster shifts, we refer to Cohen’s d effect sizes (Table 9). The large negative effect size in LDisp-GAIT (−1.22) for the PD intervention group indicates a significant reduction in lateral instability during gait initiation—a mechanical improvement. The clustering results align with this, as post-intervention states converged toward healthy individuals (Figure 4), suggesting that the observed shifts are indicative of better balance, not just change.

**Figure 8:**
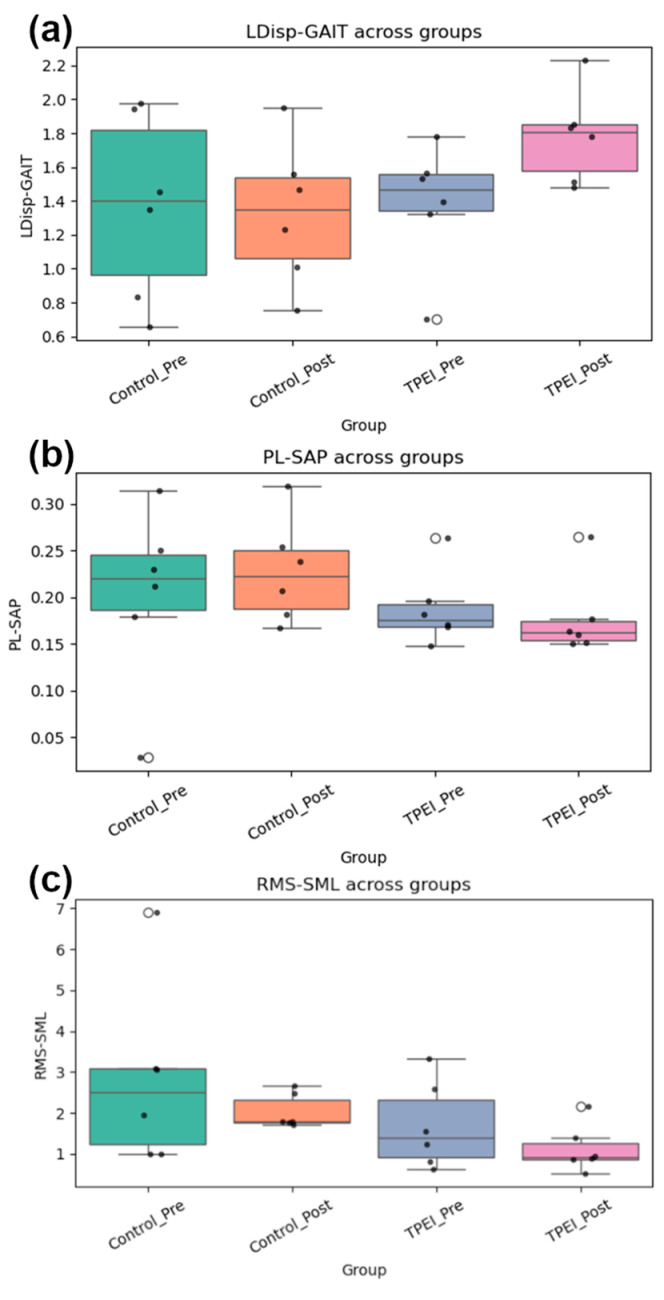
Box plots of (**a**) LDisp-GAIT, (**b**) PL-SAP, and (**c**) RMS-SML for the groups before and after the intervention are displayed.

Box plots of each feature for each group before and after the intervention are displayed in Figure 8. Also, the *p*-value was calculated through two methods (ANOVA, Kruskal–Wallis), tabulated in Table 10. No feature is significant in traditional statistical tests (*p* > 0.05). This indicates that classical statistical methods are insufficient and cannot detect significant differences before and after the intervention. This is despite the fact that our data science and clustering methods have been able to identify effective features, perform meaningful clustering, and detect distinguished patterns. Traditional statistical methods miss the information due to small sample sizes and subtle nonlinear patterns, but our machine learning approach, through feature selection and clustering, reveals post-intervention improvements. On the other hand, the algorithms were evaluated with permutation tests and nested cross-validation to check the results for randomness, overfitting, information leakage and small sample size. Also, the Cohen’s d criterion was used, and its results confirm the clustering results.

## 4. Discussion

### 4.1. Summary of Key Findings

The results suggest that all patients in the PD intervention group exhibited measurable biomechanical improvements following the intervention, as indicated by clustering analyses derived from mechanics-informed features. These features, including COP path length and root mean square deviations, depict mechanical stability and force modulation, and their changes are consistent with neuromechanical adaptations.

Specifically, patients 1, 3, and 4 of the PD intervention group in Figure 3 appear in a distinct cluster for post-intervention (blue), separate from their pre-intervention states (red). Patients 2 and 5 show a shift, with their post-intervention states appearing in different clusters than their pre-intervention states. Patient 6 similarly exhibited cluster separation, indicating a potential response to the intervention. In contrast, most patients in the PD control group remained in the same cluster (green) across pre- and post-intervention states, except for patient 2. This individual’s divergence may potentially be attributed to increased mechanical loading associated with a BMI of 33.5, which was at least six units greater than that of other participants, suggesting that anthropometric differences can influence balance mechanics. However, given the small sample size, this interpretation should be considered preliminary.

To assess the clinical significance and biomechanical relevance of these changes, we compared the post-intervention states of TPEI participants to healthy controls. As shown in Figure 4, the post-intervention states of patients 1, 3, 4, and 5 clustered with healthy individuals, indicating a possible shift toward biomechanical patterns more characteristic of healthy controls. While the distance (in the feature space) between these patients and healthy participants remained substantial, the direction of change suggests potential clinical relevance, supporting the conclusion that TPEI may contribute to restoring certain aspects of neuromechanical postural control. Notably, post-intervention data of patient 2 also moved closer to the healthy cluster, further suggesting the intervention’s potential effectiveness.

Overall, five of six TPEI patients appeared to show biomechanical improvement, whereas changes in the PD control group were minor. The PD control group participated in conventional balance, stretching, and walking exercises, which may have been less effective than the targeted transverse plane exercises in stimulating neuromuscular adaptation. Clustering analysis using both two and three clusters suggested this pattern, with slightly better clustering metrics observed for the three-cluster configuration.

### 4.2. Clinical and Practical Implications

These findings underscore the potential of force-plate data as a simple, non-invasive, and cost-effective tool for assessing motor function in PD. Unlike MRI or EMG, force-plate analysis is accessible, easy to implement in clinical settings, and well tolerated by patients. Our study suggests that even without advanced neuroimaging, meaningful insights can be obtained into the impact of rehabilitation interventions.

While Deep Brain Stimulation (DBS) is an established treatment for Parkinson’s disease, particularly for managing motor fluctuations and tremors, it has notable limitations. In addition to the inherent surgical risks, DBS is typically reserved for patients who meet strict clinical criteria, such as having levodopa-responsive symptoms and no significant cognitive impairment. Furthermore, DBS may be less effective in addressing axial symptoms such as postural instability and gait disturbances, which are often resistant to both pharmacologic and surgical interventions. This underscores the need for alternative, non-invasive therapies—such as targeted exercise programs or biomechanics-informed approaches—that directly address axial motor deficits and can be applied more broadly across the PD population.

Finally, this preliminary study demonstrates that artificial intelligence models, when combined with accessible biomechanical data, can support clinicians in both diagnosing PD and monitoring treatment efficacy. The force-plate tests used here were safe, non-invasive, and easy to administer, making them highly suitable for routine clinical use. Future research should explore the integration of additional data modalities, including EMG and neuroimaging, to further elucidate the relationship between biomechanical and neural adaptations in Parkinson’s disease. As a complementary study, a shoe embedding a load cell assessing the ground reaction force and center of pressure in standing and gait conditions can be used to record the data, and the results can be compared with a force plate [42]. It is also possible to monitor the severity of Parkinson’s disease symptoms and the patient’s overall condition in real-time by developing AI-based analysis wearable signals and integrating them with smartphones [43].

### 4.3. Comparison with Prior Works

Unlike most previous studies that used supervised classification on engineered features (speech, writing, movement, brain scan images, etc.), our proposed approach uses unsupervised clustering to detect intervention effects in Parkinson’s disease. This method uncovers hidden data structure without the need for labeled states. Compared to hierarchical clustering used for UPDRS adaptation, our clustering approach is mechanics-based and applied to COP features derived from the force plate. Furthermore, our pipeline—which includes nested cross-validation, bootstrapping, permutation testing, and SHAP analysis—ensures both statistical robustness and clear insight into feature importance. This combination of unsupervised learning with statistical analysis provides a nuanced, clinically relevant, and methodologically superior assessment of intervention effects.

Statistical analyses were performed to evaluate the differences between pre- and post-intervention states within each cluster, including effect sizes and variability measures. These analyses support the observation that TPEI participants exhibited measurable biomechanical improvements, with distinct shifts toward patterns seen in healthy controls. While the sample size remains small, these statistics provide preliminary quantitative evidence for the effectiveness of the intervention and complement the unsupervised clustering results.

### 4.4. Limitations

Several limitations of this study should be acknowledged, as they may influence the interpretation and generalizability of our findings. This study included only 12 individuals with Parkinson’s disease and 6 healthy controls. Recruitment and testing were constrained by the COVID-19 pandemic, limiting access to participants and resulting in a cohort that may not fully represent the broader Parkinson’s population. Moreover, strict inclusion/exclusion criteria were applied to avoid confounding biomechanical factors, which further restricted the number of participants. While the sample was sufficient to demonstrate the feasibility of our mechanics-informed machine learning approach, the small size may limit generalizability of the results, highlighting the preliminary nature of this investigation.

Another limitation is that the healthy control group was younger than the PD groups. Since age influences postural stability and some force-plate–derived parameters, this mismatch may partially affect the observed separation between healthy individuals and PD patients. However, this factor does not undermine the main conclusions of our study for two reasons. First, the core findings rely primarily on within-group longitudinal changes in the PD cohort (pre- vs. post-intervention), not on direct comparisons with the control group. Second, the machine-learning model integrates multiple biomechanical features that are sensitive to motor impairment rather than age alone. Even so, future work will include a wide age range of the control group to remove this potential confounder and to improve generalizability.

Although participants in the PD intervention group performed structured TPEIs at home, variability in adherence, execution, and environmental factors may have influenced outcomes. Weekly video monitoring and mid-study visits were implemented to reduce variability, but some inconsistency is inevitable in home-based protocols.

We did not include commonly used clinical outcome measures such as the Unified Parkinson’s Disease Rating Scale (UPDRS) or Timed Up and Go (TUG). While our study prioritized objective, instrumented measures to detect subtle biomechanical changes, this limits direct comparison to conventional clinical assessments. However, clinical scales may lack the sensitivity needed to detect early or minor motor impairments, which force plate-derived metrics (e.g., COP, RMS-SML, PL-SAP) can potentially capture more precisely. Future work should integrate these clinical scales alongside biomechanical features to enable correlation analyses, enhance predictive validity, and support the development of hybrid tools that improve clinical decision-making and monitoring in Parkinson’s disease.

Due to the above constraints, this study should be interpreted as a preliminary, proof-of-concept investigation. While results indicate promising trends and support the feasibility of the proposed methodology, larger, multi-center studies with diverse participants are required to confirm effectiveness and reproducibility. By explicitly recognizing these limitations, we aim to contextualize the findings and guide future research in the integration of biomechanics and machine learning for Parkinson’s disease assessment. Therefore, we believe that our approach complements, rather than replacing the main clinical scales.

### 4.5. Biomechanical Interpretation

From the perspective of biomechanical systems, balance control in the human body works much like a feedback loop which responds to shifts in position and force using principles grounded in Newtonian mechanics. When patients show better control over their COP after the intervention, it suggests that they have become more capable at adjusting themselves in response to small disturbances and redistributing forces through their feet and legs. In practical terms, this means they better become stable, which preliminarily indicates the intervention helped them improve how internal forces are coordinated to maintain balance during movement.

From a neuromechanical and physiological perspective, transverse plane exercises may enhance motor performance by improving muscle activation, neuromuscular control, and proprioception. These movements likely stimulate mechanoreceptors, including muscle spindles and Golgi tendon organs, which play a critical role in balance and coordination. Improvements in central nervous system function—such as increased motor cortex activity and improved corticospinal tract signaling—may also contribute to the observed biomechanical changes. These effects support the role of mechanotransduction in rehabilitation and highlight the importance of sensorimotor integration in PD recovery.

From Table 9, RMS-SML appears to show the clearest clinical effect. The medium to large effect size for the PD intervention group before and after the intervention suggests that the intervention may have contributed to improvement in this COP measure. In the control group, there are few or no systematic changes observed. Therefore, according to the PL-SAP and LDisp-GAIT, any effect appears negligible. RMS-SML has a moderate effect, and due to study limitations, it cannot be explicitly stated what the cause may occurred; however, what is clear and can be emphasized is the greater improvement of the PD intervention group members. Nevertheless, they remain distinct from healthy individuals. According to Table 9, large negative Cohen’s d values (−4.5, −12.1) indicate that even after the intervention, patients still have significant differences from healthy individuals. This suggests that the intervention improves COP measures but does not completely normalize them as from clinically perspective, physiotherapy rarely eliminates all disorders.

### 4.6. Feature Relevance and Validation

Figure 7 shows that RMS-SML and PL-SAP contribute the most to distinguishing PD patients from healthy individuals. These converge with the effect size analysis and reinforce the clinical significance of these features in describing improvements in postural stability. This convergence underscores that our AI-based findings are not only statistically robust but also potentially clinically interpretable and acceptable.

The top features identified—RMS-SML, PL-SAP, and LDisp-GAIT—are strongly linked to core motor deficits in Parkinson’s disease, particularly those affecting postural stability and fall risk. RMS-SML reflects medio-lateral sway amplitude and is a known marker of lateral instability, which is exacerbated in PD due to impaired axial coordination and delayed compensatory responses [44]. PL-SAP quantifies anterior–posterior sway effort and is often elevated in PD due to overcompensation or inefficient balance strategies [45]. LDisp-GAIT captures dynamic lateral movement during gait initiation, directly tied to anticipatory postural adjustments (APAs), which are frequently disrupted in PD.

These interpretations align with prior literature and our own recent studies on proprioceptive and balance rehabilitation in PD [46,47]. Unlike earlier works, this study combines biomechanics with explainable AI tools (e.g., SHAP), offering new insight into feature-level importance and supporting the use of these metrics as potential digital biomarkers for treatment monitoring.

Our study focused on force-plate-derived biomechanical signals due to their high precision in quantifying postural control metrics (e.g., center-of-pressure dynamics, sway variability), which are critical for assessing PD-related balance impairments. However, we acknowledge the growing utility of multimodal approaches (IMU-based, EMG-based, wearable gait analysis) in clinical and ambulatory settings. IMU-based systems offer portability and continuous monitoring but may lack the spatial resolution of force plates for fine-grained postural analysis [48]. EMG-based methods provide neuromuscular insights but require additional signal processing to correlate muscle activation patterns with balance performance [49]. Wearable gait analysis (e.g., inertial sensors) is practical for long-term monitoring but typically focuses on temporal-spatial parameters rather than the kinetic details captured by force plates [50].

While our validation (nested cross-validation, bootstrapping, and permutation testing) mitigates overfitting risk, future work should also explore data augmentation techniques to enhance robustness. Recent advances in generative modeling and synthetic data augmentation have shown promise in rare gait disorders, where sample availability is inherently limited [51]. Neural networks can also be utilized for analysis to develop models, in order to make the feature extraction and classification process more stable and reliable [52].

## 5. Conclusions

In this preliminary study, we explored a mechanics-informed machine learning approach that leverages non-invasive force-plate assessments to identify patterns associated with Parkinson’s disease and evaluate the effects of rehabilitation. By integrating this biomechanical data as outputs of a mechanical system governed by Newtonian dynamics with machine learning models, we demonstrated the feasibility of using a simple and non-invasive pipeline to uncover patterns related to postural control and motor function in PD. This accessible and clinically feasible approach offers meaningful insights with minimal burden on patients and clinicians.

The main findings of this exploratory study are as follows:

(1) TPEI appeared to lead to measurable improvements in postural control and motor function in the PD intervention group.
(2) RMS-SML and PL-SAP were identified by SHAP analysis as the most influential features distinguishing PD patients from healthy individuals.
(3) Clustering results suggested that post-intervention PD participants exhibited biomechanical patterns shifting toward the healthy group.
(4) Mechanics-informed features, combined with dimensionality reduction and clustering, provided physiological interpretable insights that align with clinical observations.
(5) Despite the small sample size, which needs to be considered as a main limitation of this study, the consistency across clustering, SHAP, and effect size analyses supports the feasibility of the proposed approach as a proof-of-concept framework.

Our findings indicate that transverse plane exercises may influence postural dynamics in the PD intervention group, resulting in measurable improvements in balance and motor function and consistent shifts in their biomechanical signatures. When compared with healthy individuals, certain clustering patterns appeared to reflect movement toward more stable postural strategies. These results suggest that the exercise may have improved the ability of the patients to regulate the COP dynamics and redistribute ground reaction forces. While motor and balance symptoms in PD are often clinically observable, this quantitative, force-plate-based strategy offers potential for early diagnosis, continuous monitoring, and the evaluation of physiotherapy-based interventions. Rather than relying on conventional classification metrics, our analysis focused on unsupervised clustering, dimensionality reduction, and their statistical analysis. While traditional statistical tests failed to identify significant changes, the combination of feature selection, clustering, SHAP values, and Cohen’s d effect sizes suggests that data-driven, mechanics-informed models can complement existing clinical evaluation methods.

While the results of this exploratory study are promising, they must be interpreted cautiously due to the small sample size (12 PD patients, 6 healthy controls), which limits statistical power and generalizability. Intra-subject variability, particularly in home-based interventions with variable adherence and environmental conditions, may have influenced outcomes. Although nested cross-validation, bootstrapping, and permutation testing were employed to reduce the risk of overfitting, this risk is not eliminated given the limited cohort. Moreover, standard clinical scales such as UPDRS and TUG were not included, limiting direct comparison with conventional assessments.

Looking forward, increasing the sample size, incorporating longitudinal data, and aligning outcomes with standard clinical scales will enhance the robustness of this framework. Additionally, future studies could benefit from the development of computational simulations for Parkinson’s disease progression using force-plate data as a primary input. These models could help predict individual responses to different therapeutic interventions, offering a pathway to personalized care. While the current approach prioritizes simplicity and clinical feasibility, combining force-plate data with complementary modalities—such as neuroimaging, EMG, or genetic information—can deepen our understanding of the disease’s underlying mechanisms while maintaining clinical practicality. The current work aimed to evaluate intervention effects at two time points (pre- and post-rehabilitation) using force plates, as this allowed rigorous quantification of short-term biomechanical changes. However, continuous monitoring (e.g., via wearables) would require longitudinal data collection and dynamic feature extraction (e.g., sliding-window analysis). We emphasize that our framework is not inherently restricted to force plates; its mechanics-informed design (e.g., prioritizing dynamic stability features) could inform wearable-based PD models with further validation. Future studies should integrate standard clinical scales to better align ML-derived metrics with established clinical benchmarks.

Overall, our exploratory study contributes to the emerging intersection of biomechanics and machine learning, opening up promising directions for a low-cost and scalable solution toward diagnosing PD and evaluating intervention outcomes. While still in early stages, this method may help bridge the gap between clinical needs and technological accessibility, support the personalized rehabilitation, and contribute to the development of mechanics-informed care strategies in real-world healthcare settings.

## Figures and Tables

**Table 1:** Summary of studies on biomechanical feature analysis and machine learning approaches for Parkinson’s disease diagnosis, monitoring, and intervention assessment.

Ref./Pub. Year	Objective: Physical Intervention Data Type	Participant	Method	Goal	Results
[29]/2005	Treadmill Walking COP Metrics	66 PD (30 control)	Statical → *p*-value	Intervention impact Evaluation	Improved walking speed Increased Path length
[30]/2005	Treadmill Walking COP Metrics	18 PD	Statical → *p*-value	Intervention impact Evaluation	reduction in falls and improvements in gait and dynamic balance
[31]/2015	Treadmill Walking COP Metrics	18 PD	Statical → *p*-value	Intervention impact Evaluation	Improve gait speed and stride length
[32]/2018	Yoga Questionnaires	40 PD (20 control)	Statical → *p*-value	Intervention impact Evaluation	improving motor function in PD
[33]/2020	No intervention. Electroencephalogram(EEG)	10 PD + 5 HI	MLP	Classification of HI vs. PD	Accuracy: 0.965 F1 score: 0.976 Recall: 0.970 Precision: 0.955
[34]/2023	speech signal analysis	28 PD	SVM CNN	Improving PD recognition	High F1-score High accuracy High sensitivity
[35]/2022	MRI	305 PD +227 HI	CNN	Potential to distinguish patients with PD	High accuracy
[36]/2024	Transcranial Sonography	854 PD + 775 HI	DCNN	Developing a diagnostic approach	Improving accuracy in Diagnosing
[37]/2021	wearable-derived features of gait and mobility	332 PD + 100 HI	Random Forest Decision Tree neighborhood components analysis	To discriminate stages of PD & controls vs. PD	Augment monitoring of disease progression

**Table 2:** Demography of Patients. BMI: Body Mass Index (kg/m^2^).

BMI	Age	Sex	ID
24.8	59	Female	C01
25.5	70	Male	C02
26	76	Male	C03
26	69	Male	C04
23.4	79	Female	C05
27.5	56	Male	C06
23.2	47	Male	E01
33.5	60	Male	E02
18.3	40	Male	E03
26.8	47	Male	E04
18.7	36	Female	E05
24.9	75	Male	E06
20.52	22	Male	H01
23.44	23	Female	H02
23.34	23	Female	H03
30.12	24	Male	H04
21.77	24	Female	H05
23.51	26	Male	H06

**Table 3:** A summary of the demographic and clinical characteristics of the participants across the three groups. BMI: Body Mass Index (kg/m^2^).

Features	PD-Control	PD-TPEI	Healthy Control	*p*-Value (PD Control vs. TPEI)	*p*-Value (PD vs. Healthy Control)
**H&Y**	2.4 ± 0.41	2.083 ± 0.2	-	0.219	-
**BMI**	25.53 ± 1.37	24.23 ± 5.65	23.78 ± 3.32	0.605	0.547
**AGE**	68.17 ± 9.11	50.83 ± 14.39	23.67 ± 1.37	0.056	<0.001
**SEX (Male\Female)**	4\2	5\1	3\3	-	-

**Table 4:** Key Mechanically Derived Features Selected by FFS.

Test Type	Feature Description	Symbol	Mechanical Interpretation	Model
Standing	Path length (anterior–posterior direction)	PL-SAP	Postural sway energy; instability indicator	KNN
Gait Initiation	Displacement of COP during loading phase	LDisp-Gait	Neuromechanical readiness to initiate movement	LR
Standing	Root Mean Square of COP (mediolateral direction)	RMS-SMl	Mechanical noise or oscillation in balance control	LR KNN

**Table 5:** Statistical metrics for clustering results of Figure 3.

k = 3				
No. Cluster	Mean	Variance	Standard Deviation	Size
**1-Green**	[−90.97, −19.81]	[3019.45, 8156.7]	[54.95, 90.31]	14
**2-Red**	[47.62, 292.91]	[695.79, 386.77]	[26.38, 19.67]	5
**3-Blue**	[189.99, −105.34]	[291.49072, 273.51746]	[17.0731, 16.538363]	5
**k = 5**				
**1-Green**	[−58.37, −117.59]	[208.99, 887.38]	[14.46, 29.79]	6
**2-Red**	[−80.19, 56.52]	[8.29, 1363.11]	[2.88, 36.92]	6
**3-Blue**	[189.99, −105.34]	[291.49, 273.52]	[17.07, 16.54]	5
**4-Yellow**	[47.62, 292.91]	[695.79, 386.77]	[26.38, 19.67]	5
**5-Light Blue**	[−221.08, 44.56]	[17.55, 41]	[4.19, 6.48]	2

**Table 6:** Statistical metrics for clustering results of Figure 4 and Supplementary Figure S1.

k = 2				
No. Cluster	Mean	Variance	Standard Deviation	Size
**1-Green**	[−83.12, −38.48]	[1103.26, 9483.75]	[33.22, 97.38]	13
**2-Red**	[71.9, 54.81]	[948.67, 4896.98]	[30.8, 69.98]	13
**k = 3**				
**1-Green**	[71.9, 54.81]	[948.67, 4896.99]	[30.8, 69.98]	17
**2-Red**	[−120.59, 83.48]	[29.91, 113.35]	[5.47, 10.65]	5
**3-Blue**	[−59.7, −114.7]	[348.33, 233.63]	[18.66, 15.28]	8
**k = 4**				
**1-Green**	[106.69, 133.33]	[75.57, 358.98]	[8.69, 18.95]	7
**2-Red**	[47.55, −0.1536]	[119.6, 737.1]	[10.94, 27.15]	10
**3-Blue**	[−120.59, 83.48]	[29.91, 113.35]	[5.47, 10.65]	5
**4-Yellow**	[−59.7, −114.7]	[348.33, 233.63]	[18.66, 15.28]	8

**Table 7:** Statistical metrics for clustering results of Figure 5.

No. Cluster	Mean	Variance	Standard Deviation	Size
**1-Green**	[67.98, 12.24]	[432, 144.08]	[20.79, 12]	11
**2-Red**	[−100.05, −33]	[44.61, 106.29]	[6.68, 10.31]	17

**Table 8:** The performance of the K-Means clustering algorithm for intervention evaluation on unseen data and validation through bootstrapping.

No. of Cluster	Silhouette	Davies–Bouldin	Calinski–Harabasz
2 95% CI	0.791 [0.7008, 0.8710]	0.45 [0.1026, 0.8412]	100.828 [28.8638, 296.2418]
3 95% CI	0.770 [0.54734, 0.8759]	0.294 [0.0883, 0.5617]	184.920 [95.1136, 329.6973]

**Table 9:** Cohen’s d effect sizes for COP characteristics under pre- and post-intervention conditions for both groups.

Feature	TPEI Pre vs. Post-Intervention	Control Pre vs. Post-Intervention	Healthy vs. TPEI Post-Intervention
PL-SAP	0.24 (Small)	−0.32 (Negligible)	−4.53 (Huge)
RMS-SML	0.66 (Medium Large)	0.51 (Medium)	−12.1 (Huge)
LDisp-GAIT	−1.22 (Large)	0.08 (Negligible)	0.33 (Small)

**Table 10:** *p*-values for three features are calculated.

Feature	ANOVA	Kruskal
PL-SAP	0.559	0.236
RMS-SML	0.16	0.0956
LDisp-GAIT	0.232	0.256

## Data Availability

Data generated during the current study are available from the corresponding author on reasonable request.

## Supplementary Materials

The following supporting information can be downloaded at: https://www.mdpi.com/article/10.3390/diagnostics15222855/s1, Figure S1: Dimensionality reduction of data obtained from the healthy individuals and patients using t-SNE with a perplexity of 3 is illustrated.

## List of Abbreviations

**Abbreviation**	**Full Form**
PD	Parkinson’s Disease
ML	Machine Learning
COP	Center of Pressure
t-SNE	t-Distributed Stochastic Neighbor Embedding
DBS	Deep Brain Stimulation
PI	Proprioceptive Intervention
TPEI	Transverse Plane Exercise Intervention
KNN	K-Nearest Neighbors
SVM	Support Vector Machine
FNN	Feedforward Neural Network
MRI	Magnetic Resonance Imaging
RF	Random Forest
PCA	Principal Component Analysis
EEG	Electroencephalography
EMG	Electromyography
ANN	Artificial Neural Network
PL-SAP	Path Length—Sway Anterior–Posterior
RMS-SML	Root Mean Square—Sway Medio-Lateral
LDisp-GAIT	Lateral Displacement—Gait Initiation
H&Y	Hoehn and Yahr (Parkinson’s stage scale)
BMI	Body Mass Index
FMCOP	Force, Moment, and Center of Pressure
FFS	Forward Feature Selection
RFE	Recursive Feature Elimination
EFS	Exhaustive Feature Selection
DBSCAN	Density-Based Spatial Clustering of Applications with Noise
KL	Kullback–Leibler Divergence
SFV	Selected Feature Vector
WCSS	Within-Cluster Sum of Squares
SD	Standard Deviation
UPDRS	Unified Parkinson’s Disease Rating Scale
TUG	Timed Up and Go
APA	Anticipatory Postural Adjustment
