# Supplementary material for: A Preliminary Mechanics-Informed Machine Learning Framework for Objective Assessment of Parkinson’s Disease and Rehabilitation Outcomes
Source: Diagnostics (Basel). 2025 Nov 12;15(22):2855. doi: 10.3390/diagnostics15222855 (PMC12651874; doi:10.3390/diagnostics15222855)
Supplement: Supplementary file 1 [file diagnostics-15-02855-s001.zip › diagnostics-3883062-supplementary.pdf]

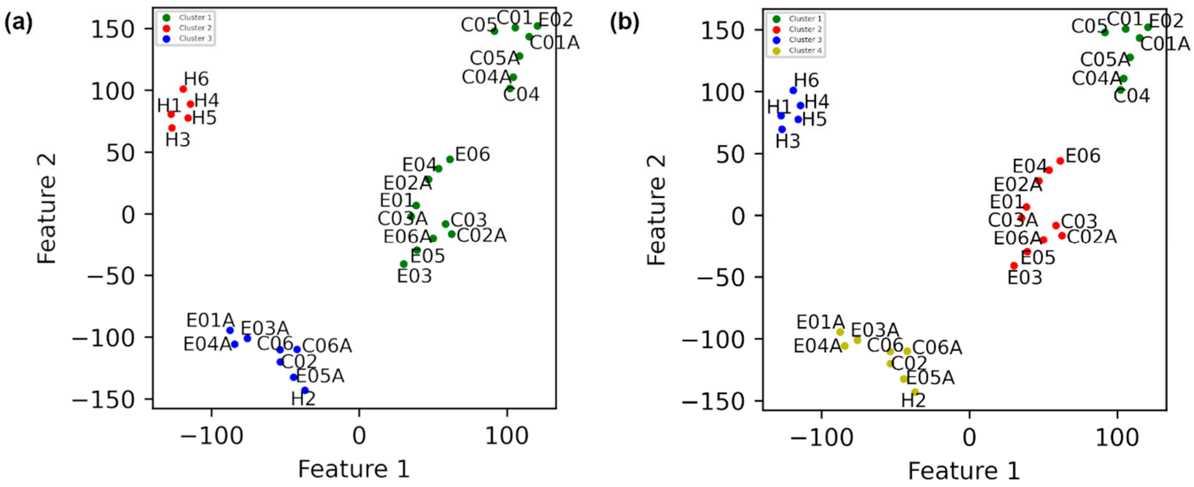


**Supplementary Figure S1.** Dimensionality reduction of data obtained from the healthy individuals and patients using t-SNE with a perplexity of 3 is illustrated. (a) K-Means clustering with k = 2. (b) K-Means clustering with k = 3. The labels E, C, A, and H in the names denote with intervention exercise, control, after exercise, and healthy individuals, respectively.
